# What can health care professionals in the United Kingdom learn from Malawi?

**DOI:** 10.1186/1478-4491-7-26

**Published:** 2009-03-27

**Authors:** Ron Neville, Jemma Neville

**Affiliations:** 1 Westgate Medical Practice, Dundee, UK; 2 Communications Manager, Edinburgh, UK

## Abstract

Debate on how resource-rich countries and their health care professionals should help the plight of sub-Saharan Africa appears locked in a mind-set dominated by gloomy statistics and one-way monetary aid. Having established a project to link primary care clinics based on two-way sharing of education rather than one-way aid, our United Kingdom colleagues often ask us: "But what can we learn from Malawi?" A recent fact-finding visit to Malawi helped us clarify some aspects of health care that may be of relevance to health care professionals in the developed world, including the United Kingdom. This commentary article is focused on encouraging debate and discussion as to how we might wish to re-think our relationship with colleagues in other health care environments and consider how we can work together on a theme of two-way shared learning rather than one-way aid.

## Introduction

Health is global. Health is local. Health is individual. How should health care professionals conceptualize and then act on the need to manage their individual patients as best they can in a local and personal context, while taking account of the globalization of many aspects of society, including health? How can we solve the conundrum of acting both locally and globally to help patients?

Health has become a global issue because viruses such as HIV and SARS do not respect international borders [1,2]. The failure of immunization delivery in one country can precipitate the return of a disease in surrounding countries, as the recent example of polio in Nigeria shows [3]. Malaria eradication schemes in one area are ineffective unless neighbouring countries adopt similar strategies [4].

Access to uncontaminated water is a fundamental prerequisite for good health. Conflict zones, water supply disputes and lack of engineering infrastructure prevent large areas of the world's population from achieving a reasonable health status [5].

Availability of trained health care staff is inequitable across urban and rural areas, across borders and regions. Staff recruiting policy in one country can destabilize health care provision in another country. For example, the United Kingdom's importing nurses to support the expansion of care homes for the elderly led to a further exodus of trained nursing staff from southern Africa [6].

The availability and supply of modern pharmaceuticals has become an issue of international concern, particularly with regard to antiretroviral HIV drugs. Seemingly simple solutions to global inequality of supply, such as the free provision of high-quality pharmaceuticals manufactured in the developed world, have been blighted by the reappearance of the same drugs sold back into the developed world and also by the appearance of poor-quality counterfeit products [7].

The emergence of the Internet as the dominant source of educational support in both the developed and the developing world has fostered a culture and understanding of global health issues [8]. Social networking and more formalized institutional links are gradually raising awareness of the need to appreciate the similarities and shared ambitions of health professionals worldwide. Many professionals now have experience of working in more than one country and have an unmet aspiration to work and help their colleagues in other health care environments.

The old-fashioned model of "colonial aid" or project-specific nongovernmental organization (NGO) work based on a "donor and recipient" model is becoming discredited. One-way aid donation is sometimes not only ineffective, it can have a detrimental effect. In resource-poor countries, one-way aid can encourage dependence, present an opportunity for corruption, replicate systems of inappropriate training and encourage urbanization of resources and personnel, leading to emigration of trained personnel [9].

Increasing cooperation between different health care systems should be a two-way dialogue of support rather than a one-way flow of aid. Just as technology companies can learn practical solutions emerging from resource-poor countries, so too can health care professionals learn from their colleagues. Increased opportunity for travel and networking by means of the Internet and low-cost telephone options have raised awareness of the potential for health care professionals in different environments to share experiences, learning and skills. Such learning and sharing have the potential to help affluent countries just as much as resource-poor ones.

The establishment of a project to twin Scottish general practices with Malawian clinics and a recent fact-finding visit prompted our colleagues to challenge us on the assertion that learning can be two-way [10]. This article outlines some of the areas where health care professionals in the United Kingdom can learn from Malawi.

## Discussion

### Structure of health care

Both Scotland and Malawi enjoy almost universal access to primary care, free at the point of delivery. Both countries have a well-developed structure of primary care. In the United Kingdom, this is based around the National Health Service and the general practitioner (GP) list system. In Malawi, Health Ministry clinics provide a backbone of primary, public health and maternity services in urban townships and rural population centres. There is a modest level of private health care provision and a substantial contribution from various NGOs – largely of a thematic nature – for example HIV, malaria or tuberculosis (TB) care. The Health Ministry has a very clear policy for health care delivery, including public health. There is a well-organized structure of district health offices (DHO) with a chain of command, although not always accompanied by the logistic or fiscal capability to implement policy.

### Medical records

The medical records system in the United Kingdom, despite an investment of several billion pounds sterling in hardware and software support, remains fragmented and inefficient [11]. A rather turgid debate rumbles on about whether patients can be trusted to hold or gain access to their records [12]. In Malawi all citizens are issued with a "health passport" for a token fee. This is a small paper booklet with customized versions for children and women of childbearing age (Additional file 1).

The health passport is treated with reverence, but some copies succumb to loss, falling in the fire or getting wet in the rainy season. Many Malawians store the health passport in the plastic bags commonly used for weighing sugar. The booklet provides a complete and integrated record of immunizations, preventive health care priorities, major medical morbidities and a continuation record of clinical encounters.

In the United Kingdom we could learn from this experience by taking patient responsibility for records systems seriously, whether paper or electronic. We could also learn to use an integrated record to encompass primary and secondary care and preventive health care. Fundamentally, taking steps to further involve patients in the management of their own health care records will increase the perception of transparency in the clinician/patient relationship and maintain trust.

### Guardianship project

The Malawian health care system has to prioritize which patients are suitable for expensive treatments, including antiretroviral therapy for HIV or anti-TB therapy. In order to be accepted on to a treatment programme, patients have to give a commitment to try to maintain their nutrition, take their medication as directed and be present for follow-up appointments. To facilitate these, patients must nominate a "guardian" – usually a close family member. The guardian must then accept responsibility for ensuring that the patient takes the right sort of nutritious food, checks medication compliance and makes sure the patient is present for follow-up. Some clinics even provide a shelter and cooking facilities for the guardians [13].

In the United Kingdom it would of course be unacceptable to deny patients treatment because they were unable to produce a guardian or make a promise to comply with therapy, but there are lessons to be learnt from the Malawian policy. Patients in the United Kingdom requiring treatment for HIV, hepatitis B or C or TB also need to maintain good nutrition, comply with medication regimes and be present for regular follow-up. A voluntary guardian system might well be acceptable to United Kingdom patients and have the potential to favourably alter clinical outcome. It is the norm in antenatal and intrapartum care for United Kingdom patients to be accompanied by a "guardian" (usually their partner). Supportive partners may welcome an opportunity to become more involved in care and may be receptive to being given enhanced responsibility.

Perhaps the care of people with long-term health conditions is where the guardianship model could be most useful. The clinical outcome in people with diabetes, arthritis, ischaemic heart disease and chronic obstructive pulmonary disease (COPD) depends on good nutrition, medication compliance and follow-up care according to management guidelines [14]. Again the experience of mentoring suggests that developing and formalizing support for patients may improve outcome. There is research evidence to suggest that clinical outcomes in cancer care can be enhanced if patients are "mentored": supported by volunteers or fellow sufferers [15]. This support could be formalized to include a "guardian" commitment to provide good nutrition and assist with medication compliance and follow-up appointments. It would be interesting to tease out the relative contribution that shared responsibility and family support make to clinical care in the resource-rich developed world.

### Direct link between public health and clinical care

With the decline of infectious diseases in the developed world due to improved hygiene, provision of fresh water and improved nutrition, less emphasis is now placed on public health initiatives. There is an apparent disconnect between care providers and those engaged in public health. Very few front-line health care professionals in the United Kingdom perceive themselves as having a direct public health role. The situation in Malawi is very different, because of the high and visible presence of infectious diseases affecting the population. GPs and midwives in the United Kingdom might wish to re-learn outreach and public health skills. In Malawi, clinical officers make direct and immediate contact with their public health outreach colleagues if they suspect a water source is contaminated or an open case of TB is present in the community. In the United Kingdom, we might wish to reconsider how health professionals use their atrophied public health skills to tackle preventable problems such as smoking, excess alcohol consumption, poor dietary habits and lack of exercise [16].

An accepted task of Malawian midwives and clinical officers is to teach groups of patients about important public health matters such as condom use, good nutrition and obtaining fresh water. This teaching can include a dance or a song in keeping with local or village custom of conveying a message to peers. Patients in the United Kingdom would certainly remember a smoking cessation advice session that included singing and dancing with their doctor or nurse. While not advocating that the NHS provide dancing sessions en masse, the message is clear. Engaging with local communities requires the use of communications media tailored to the target audience.

### Voluntary counselling and testing (VCT)

In the United Kingdom, one in three HIV-seropositive persons is unaware of his or her status [17]. Despite public health efforts to increase the uptake of testing, and despite the availability of retroviral therapy, testing for HIV still carries a stigma in the United Kingdom health system. Many patients are reluctant to turn to mainstream health facilities, such as their own GP, and turn instead to more secretive and less personal forms of care, such as genitourinary medicine clinics. In Malawi, HIV testing is available in Health Ministry clinics and is voluntary, linked with counselling, hence VCT. "VCT" has entered the everyday vocabulary of Malawians because clinics display signs and an ongoing poster campaign helps to dispel stigma. Crucially, testing is available on demand with the result available quickly, sometimes on the same day. It is remarkable that a developed, resource-rich health care system such as the United Kingdom's NHS still persists with the archaic practice of keeping patients waiting at least a week to receive a result. The Malawian experience of destigmatizing and simplifying on-the-spot testing may be relevant to persons at risk in the United Kingdom [18].

### Kangaroo special care baby unit

The outcome for premature babies in resource-poor countries used to be very unfavourable. In South America, the "kangaroo care" model was developed. Premature babies are placed between their mother's breasts. A woollen hat is provided to minimize heat loss through the baby's scalp, and the mother's body acts as an incubator (Additional file 2). Breast milk is expressed directly into the baby's mouth, or onto cotton wool and then squeezed gently into the baby.

"Kangaroo care" can keep vulnerable premature babies alive in environments where incubators and tube feeding are not available [19]. In Malawi, maternity units have a "kangaroo care" section. Mothers are assisted by their "guardians", usually a grandmother. They take it in turn to nurse the baby. While one is lying with the baby in the "kangaroo" position, the other prepares food and talks with other mothers and midwives. Every so often they exchange roles.

There may be aspects of kangaroo care applicable to intermediate-level, special-care baby support in the transition from high-dependency, extreme-premature incubator support to low-level support with parental involvement.

### Protocols

A feature of clinic work in Malawi is the set protocols for managing likely presenting problems. These are reminiscent of guidelines and flowcharts displayed in accident and emergency departments [20]. Perhaps all health care professionals should accept that it is good and accepted practice to constantly refer to protocols, and to share with patients an openness to consulting guidelines.

### Youth drop-in centres in clinics

A feature of Malawian health care is drop-in youth centres. Such centres are located within or close by health facilities and, unlike in the United Kingdom, are not separated and delivered by different agencies across the health and social service divide. Empowering young people through social responsibility, care for the elderly, sports and employment training is a message as relevant in Europe as in Africa [21].

### Health education seminars from student nurses

Student nurses in Malawi have to offer health educational group sessions as part of their training. This requires a high level of communication skill and an ability to cast aside inhibition to make sure an important health message is conveyed (Additional file 3). There are opportunities for Malawian nurses to share this expertise with their more reserved colleagues in the United Kingdom.

### Endless patience and tolerance

While sitting in on clinics, we were struck by the attitudes of patients and staff. Acceptance of adversity, perhaps borne out of experience of hunger or lack of resources, allowed a health care system to cope with large numbers of patients every day. It was culturally unacceptable to make a fuss and so health care staff members were able to concentrate on seeing ill people in order of clinical need rather than according to who protested the loudest or booked in first.

In a clinical system of high throughput, rapid diagnosis and lack of treatment options, complaining was seen as futile. We found the lack of privacy during consultations unsettling, particularly when two or more consultations took place in the same room or the same space outdoors. Our "prudishness" became a source of some amusement in an environment where attending to one's toileting needs was often a public matter.

### Pride in registered nursing

In Malawi, International Nurses Day is celebrated each year, complete with the "Nurses' Pledge, Prayer and Song" [22]. These feature the core values of nursing and are matters of intense pride for nurses. It is interesting to speculate whether nurses in the United Kingdom are sufficiently proud of their hard work to sing a song about it in front of their patients. Malawian clinics fly the national flag and are proud to be associated with the Ministry of Health. It is doubtful whether United Kingdom clinics feel a similar need to be associated with the NHS. Perhaps endless changes of health service administration have dampened enthusiasm for United Kingdom staff to take pride in their institutions.

### Paradox

Health care in Malawi is underresourced. Patients die needlessly due to lack of adequate medical facilities. Life expectancy is short. Resource problems mean that Malawi lags behind almost all other countries in measures of clinical outcome. But therein lies a paradox: the so-called "worse" can teach the so-called "best" many lessons. Perhaps we should look to our colleagues in Malawi and other resource-poor environments to help us reconnect with the core values of patient autonomy, simple recordkeeping, careful use of resources, adherence to protocols, innovation to tackle health education, integration of public health with clinical care and above all, professional pride in caring for patients.

The barriers to change within each health care environment appear to be very different. Clearly, increased monetary resources are needed in Malawi. Increased pay for staff, availability of medicines and equipment delivered uncritically could destabilize a system that can count organized primary care linked to public health as major assets. "Westernization" of health care has the inherent risk of promoting consumerism, urbanization and hospital care – none of which is likely to raise the health outcomes for the majority of the population, who live by subsistence agriculture based around village communities with strong family ties. The United Kingdom has recently learnt the painful lesson that increased monetary input does not directly correlate with improved health outcome. The major challenges facing the health of people in the United Kingdom are linked to lifestyle. Imaginative ways for families and communities working together to reduce smoking, increase exercise levels, improve nutrition and extend family support are more likely to yield dividends than increased GDP spent on hospitals.

## Conclusion

So what can health care professionals based in the United Kingdom and other resource-rich environments learn from Malawi? Behind the gloomy statistics and cynicism about whether one-way aid works, there is an opportunity for dialogue locally and globally. At the very least, health care professionals in the United Kingdom might want to debate and discuss what global and local health is about. Our Malawian colleagues can contribute and share in that debate. We can all learn global lessons to apply locally.

## Abbreviations

COPD: chronic obstructive pulmonary disease; DHO: district health office; GDP: gross domestic product; HIV: human immunodeficiency virus; NGO: nongovernmental organization; SARS: severe acute respiratory syndrome; TB: tuberculosis; UK NHS: United Kingdom National Health Service; VCT: voluntary counselling and testing.

## Competing interests

The authors declare that they have no competing interests.

## Authors' contributions

The idea, drafts and completion of the article are the equal work of both authors.

## Supplementary Material

Additional file 1 **Malawian health passport (in left foreground).** Click here for file

Additional file 2 **Example of kangaroo special baby care.** Click here for file

Additional file 3 **Conveying health education through song.** Click here for file

## References

[B1] Malawi national strategy for sustainable development. http://www.sdnp.org.mw/enviro/chilwa/ministry/strategicplan/nssdth5.htm.

[B2] Severe Acute Respiratory Syndrome. http://www.who.int/csr/sars/en/.

[B3] [Anonymous] (2002). Polio cases rise in Nigeria as vaccine is shunned for fear of AIDS. BMJ.

[B4] (2007). Is malaria eradication possible? Editorial. The Lancet.

[B5] Water and sanitation. http://www.waterforpeople.org/site/PageServer?pagename=Int_Malawi.

[B6] Buchan J (2002). Nurse recruitment and international migration. Nursing Enquiry.

[B7] Steinbrook R (2007). Closing the affordability gap for drugs in low-income countries. NEJM.

[B8] International medical education. http://www.gwu.edu/~intmeded/resource.html.

[B9] Horton R (2000). Development aid: manna or myth?. The Lancet.

[B10] Malawi clinics. http://www.Malawiclinics.og/.

[B11] Burke K (2002). NHS misses target for introducing electronic records. BMJ.

[B12] Al-Agilly S, Neville RG, Robb H, Riddell S (2007). Involving patients in checking the validity of the NHS shared record: a single practice pilot. Informatics in Primary Care.

[B13] Neville RG, Riddell S, Wilson P, Nkhoma P (2007). The twinning of Scottish general practices and Malawian clinics: the provision of email and internet services. Inform Prim Care.

[B14] National Institute for Health and Clinical Excellence. http://www.nice.org.uk/.

[B15] Kerr J, Engel J, Schlesinger-Raab, Sauer H, Holzel D (2003). Communication, quality of life and age: results of a 5-year prospective study in breast cancer patients. Annals of Oncology.

[B16] Public Health and Clean Water. http://www.undp.org.mw/index.php?option=com_content&view=article&id=155:access-to-clean-water-and-sanitation-a-human-rights-issue&catid=40:latest-press-release&Itemid=97.

[B17] HIV statistics in the UK. http://www.avert.org/uksummary.htm.

[B18] HIV statistics in Malawi. http://www.avert.org/aids-malawi.htm.

[B19] Kangaroo Special Baby Care. http://www.instinctiveparenting.com/flex/a_pocket_guidebook_to_kangaroo_care/77/1.

[B20] Emergency Room Guidelines. http://www.geocities.com/nyerrn/er/g.htm.

[B21] HIV and Youth in Africa. http://www.advocatesforyouth.org/PUBLICATIONS/factsheet/fshivaidsaf.htm.

[B22] International Nurses Day. http://www.icn.ch/.

