# Supplementary figures and images for: What can health care professionals in the United Kingdom learn from Malawi?
Source: Hum Resour Health. 2009 Mar 27;7:26. doi: 10.1186/1478-4491-7-26 (PMC2666626; doi:10.1186/1478-4491-7-26)

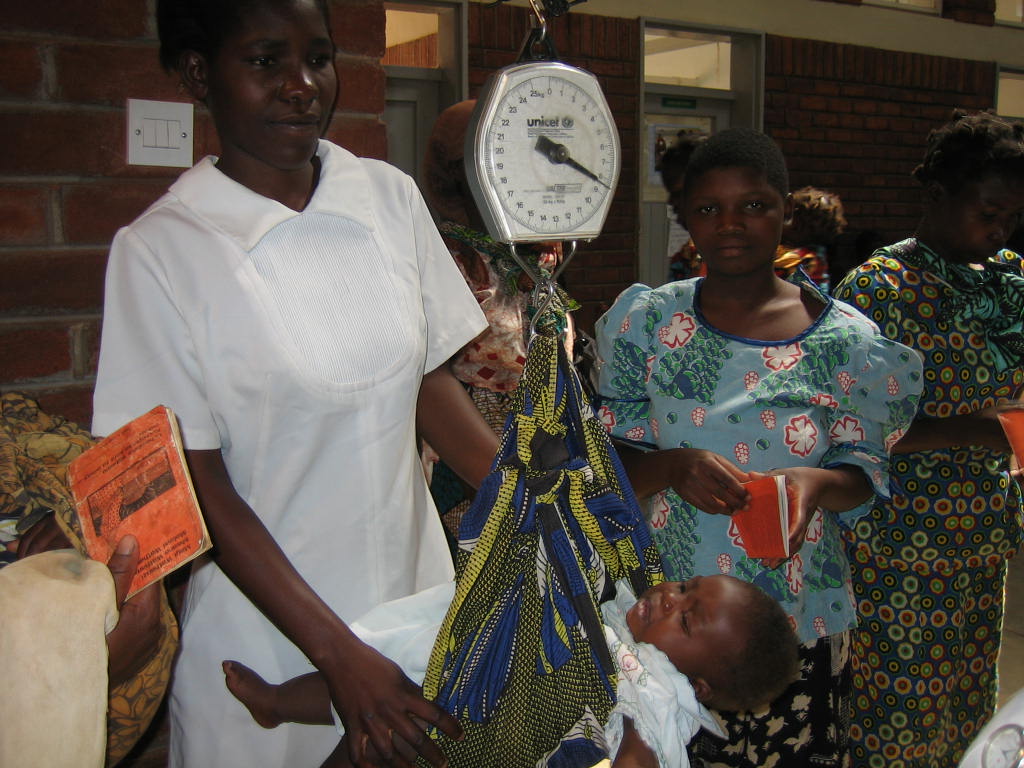

Supplement: Additional file 1 — Malawian health passport (in left foreground). [file 1478-4491-7-26-S1.jpeg]

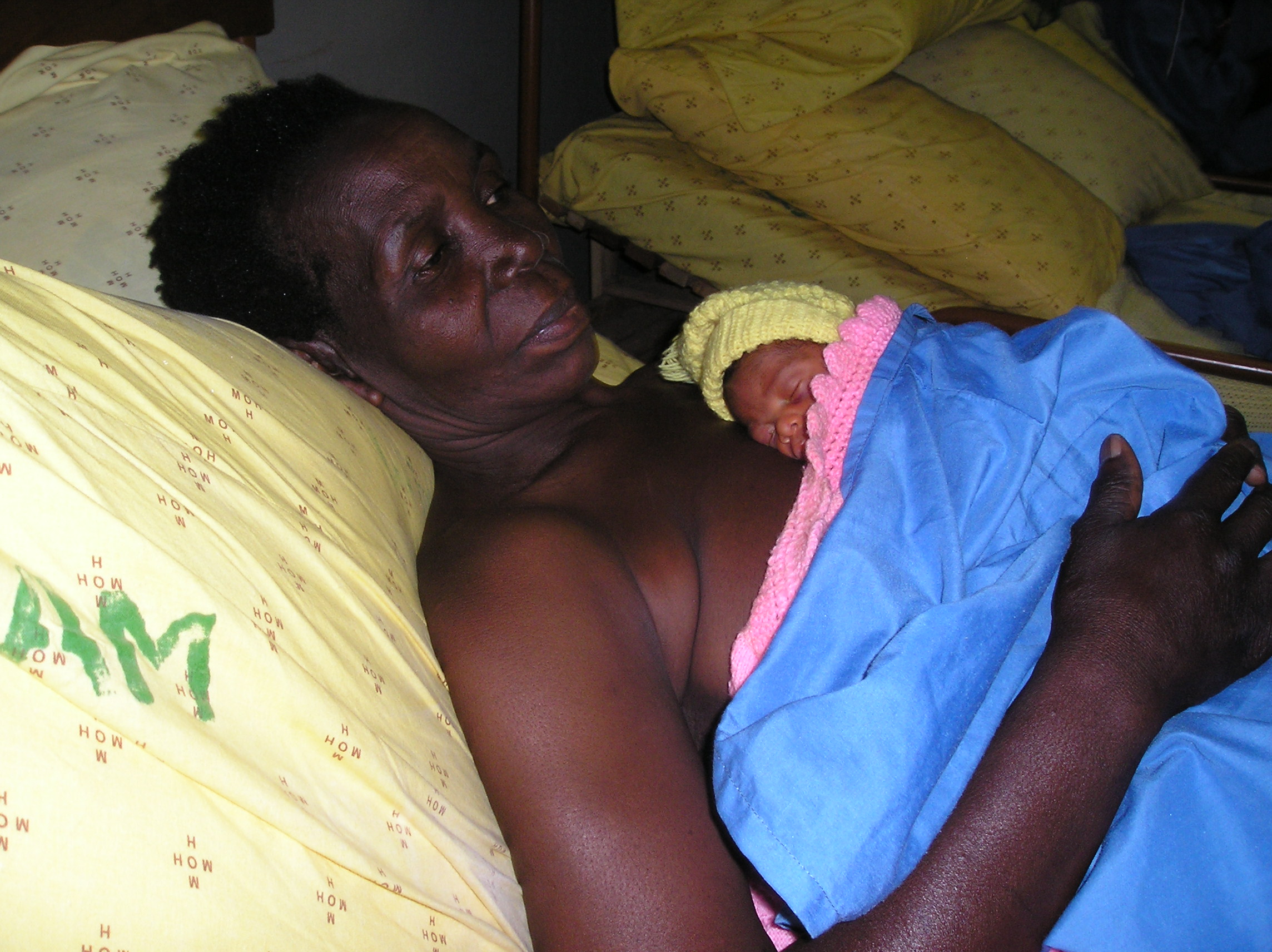

Supplement: Additional file 2 — Example of kangaroo special baby care. [file 1478-4491-7-26-S2.jpeg]

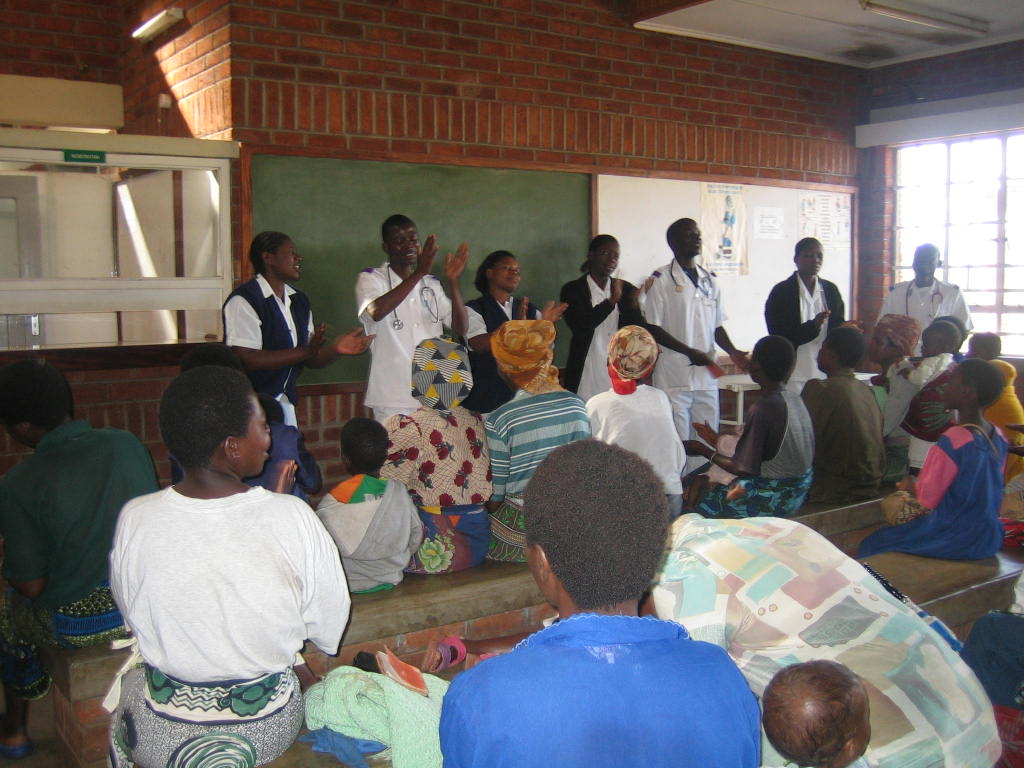

Supplement: Additional file 3 — Conveying health education through song. [file 1478-4491-7-26-S3.jpeg]
